# Using Osteopathic Medical School Institutional Data to Establish the Correlation Between Academic Performance and First-Time Board Pass Rates to Develop Academic Interventions for Students at Risk of First Board Failure

**DOI:** 10.7759/cureus.72354

**Published:** 2024-10-25

**Authors:** Kim Chosie

**Affiliations:** 1 Academic Support Services, Alabama College of Osteopathic Medicine, Dothan, USA

**Keywords:** academic, alabama college of osteopathic medicine, board preparation program, comlex, institutional data, medical school

## Abstract

The rapid increase in the number of osteopathic medical schools creates a highly competitive medical school environment as each osteopathic medical school seeks to enroll top-performing students. This results in osteopathic colleges' need to be innovative in the academic support interventions they provide to help increase their student’s chances of successfully passing their Comprehensive Osteopathic Medical Licensing Exam (COMLEX) level 1 and 2 board exams on their first attempt. A correlation analysis was performed of the Alabama College of Osteopathic Medicine’s (ACOM) class of 2024’s (188 students) pre-matriculation metrics, including Grade Point Average(GPA), Science Grade Point Average (SGPA), and Medical College Admissions Test (MCAT), performance in each course, and on two specific exams to determine if causality or correlation existed between these variables and first-time Comprehensive Osteopathic Medical Licensing Exam (COMLEX) level 1 and level 2 pass rates. Using the Spearman correlation (significant at the p<0.01 level) reveals that, if a student was in the lowest quartile in any of the following courses, they were at risk of failing COMLEX level 1: medical biochemistry (00.156), neuroanatomy (00.168), cardiology (00.275), renal (00.176), respiratory (00.235), or gastrointestinal (00.209). The lowest quartile performance in neuroanatomy (00.228) and gastrointestinal (00.154 - significant at the p<0.05 level) correlates to a first-time failure of COMLEX level 2. Specific Interventions have been developed for students in the lowest quartile of the courses identified in the findings of the institutional data analysis to better prepare them to pass the COMLEX level 1 and level 2 board exams.

## Introduction

Over the past 15 years, the number of osteopathic medical schools has increased threefold, resulting in heightened competition as institutions vie for top-performing students [1]. Consequently, many colleges of osteopathic medicine (COMs) face challenges in maintaining a consistent number of high-achieving students in each matriculating class [2]. This competitive landscape has necessitated the development of innovative academic support interventions to ensure that students remain competitive in the residency matching process, a critical milestone in their medical careers.

One of the most significant determinants of a student's success in the residency matching process is their performance on the Comprehensive Osteopathic Medical Licensing Examination of the United States (COMLEX-USA), particularly level 1 and level 2. First-time pass rates on these exams are pivotal in determining whether a student will successfully match into a residency program [3]. Therefore, ensuring students are adequately prepared for these high-stakes exams has become a top priority for medical schools.

In response to these challenges, the Alabama College of Osteopathic Medicine (ACOM) conducted a retrospective analysis of its class of 2024 to identify correlations between pre-matriculation metrics, such as grade point average (GPA), science GPA (SGPA), and Medical College Admission Test (MCAT) scores, and student performance throughout the pre-clinical curriculum [4]. Additionally, this study sought to determine whether these factors could predict first-time COMLEX-USA failures or the need for students to participate in structured board preparation programs.

By analyzing both pre-matriculation data and post-admission course performance, ACOM aimed to create a proactive roadmap for academic interventions. The goal was to improve first-time pass rates on COMLEX-USA level 1 and level 2, thus enhancing students' chances of securing a residency match and completing their graduate medical education successfully.

## Materials and methods

This retrospective cohort study using the Spearman correlation was conducted at the ACOM and involved the analysis of 188 students from the Class of 2024. The primary objective was to identify correlations between pre-matriculation metrics (GPA, SGPA, and MCAT scores) and post-admission performance with first-time failure rates on the COMLEX-USA levels 1 and 2, as well as participation in structured board preparation programs. The study also aimed to determine which pre-matriculation and pre-clinical academic variables could predict the need for academic interventions to improve board exam outcomes.

Data were collected from institutional records and included both pre-matriculation metrics (GPA, SGPA, and MCAT scores) and post-admission performance in specific pre-clinical courses. Pre-matriculation metrics were obtained from student admissions records, while course performance was derived from official grade reports for courses completed during the pre-clinical years. Additionally, first-time pass or failure outcomes on the COMLEX-USA level 1 and 2 exams were included in the analysis (IRB 24-07-18-001).

To assess the relationships between pre-matriculation metrics (GPA, SGPA, MCAT) and negative outcomes (COMLEX failures and board preparation participation), Spearman correlations were calculated. Correlations were deemed significant at the 0.01 level (2-tailed). To determine the predictive value of post-admission course performance on negative outcomes, a binary logistic regression model was employed. The dependent variables were negative outcomes (COMLEX failure or board preparation program participation), while the independent variable was quartile performance in specific courses.

Inclusion criteria for the study were students who were part of the ACOM Class of 2024 and completed the full pre-clinical curriculum, students with complete pre-matriculation data (GPA, SGPA, MCAT), students who attempted the COMLEX-USA level 1 and/or level 2 exams during the study period and students whose first-attempt pass/fail outcomes on COMLEX-USA level 1 and/or level 2 were available.

Students with incomplete pre-matriculation data (missing GPA, SGPA, or MCAT), who withdrew or transferred from ACOM before completing the pre-clinical curriculum, who did not attempt the COMLEX-USA level 1 or level 2 exams and without available first-attempt pass/fail outcomes for COMLEX-USA exams were excluded from the study.

## Results

It was determined to use data from the ACOM’s Class of 2024, which had a total of 188 graduates, as they had completed all courses and both COMLEX-USA level 1 and level 2 exams. The data included an analysis of their pre-matriculation data (GPA, science GPA, and MCAT), quartile (determined by class rank) performance in all courses, and quartile performance on anatomy exam one and medical biochemistry exam one. Findings from a review of the pre-matriculation data using the Pearson correlation (significant at the p<0.01 level, 2-tailed) reveal that lower pre-matriculation SGPA (-0.169) and MCAT (-0.205) associate weakly with students required to participate in a structured board preparation program, and higher pre-matriculation SGPA correlates weakly (0.164) with first-time pass on COMLEX-USA level 2 (Table 1).

**Table 1:** Pre-matriculation (GPA, SGPA, MCAT) risk correlates PA GPA: pre-admission grade point average; PA SGPA: pre-admission science grade point average; MCAT: medical college admissions test; COMLEX-USA: Comprehensive Osteopathic Medical Licensing Examination of the United States; USMLE: United States Medical Licensing Examination ^*^correlation significant at the 0.01 level (2-tailed); ^**^correlation significant at the 0.05 level (2-tailed); ns - correlation is not significant (2-tailed)

Board Prep Course & Board Exams	PA GPA	PA SGPA	MCAT
Boards Bootcamp	ns	-0.169*	-0.205**
COMLEX-USA Level 1: 1st Attempt Pass	ns	ns	ns
USMLE Step 1: 1st Attempt Pass	ns	0.247*	ns
COMLEX-USA Level 2: 1st Attempt Pass	ns	0.164*	ns
COMAT Failure: FM	ns	-0.182*	-0.185*

A breakdown of student performance by course and quartile using the Spearman correlation in Table 2 indicates that students who perform in the lowest quartile in any course associated with having to participate in a structured board preparation course, while performance in the endocrine and reproductive course (0.411) has the highest risk (Table 2).

**Table 2:** Risk correlates: Post-Admission Board Preparation Program COMLEX-USA: Comprehensive Osteopathic Medical Licensing Examination of the United States; USMLE: United States Medical Licensing Examination; COMAT: Comprehensive Osteopathic Medical Achievement Tests; OB/GYN: Obstetrics & Gynecology; IM: Internal Medicine; PSY: Psychiatry; SUR: Surgery; FM: Family Medicine; AS: Anatomical Sciences; MM: Molecular Medicine; Neuro: Neuroanatomy & Behavioral Sciences; Cardio: Cardiology; Resp: Respiratory; Endo/Repro: Endocrine and Reproduction; GI: Gastroenterologyorrelation significant at the 0.01 level (2-tailed) **correlation significant at the 0.05 level (2-tailed) ns - correlation is not significant (2-tailed)

Board Prep Course & Board Exams	Lowest Quartile: AS Exam 1	Lowest Quartile: AS	Lowest Quartile: MM Exam 1	Lowest Quartile: MM	Lowest Quartile: Neuro	Lowest Quartile: Cardio	Lowest Quartile: Renal	Lowest Quartile: Resp	Lowest Quartile: Endo/Repro	Lowest Quartile: GI	Lowest Quartile Total
Boards Bootcamp	0.216**	0.352**	0.310**	0.342**	0.290**	0.297**	0.254**	0.310**	0.411**	0.329**	0.533**
COMLEX-USA Level 1: 1st Attempt Pass	ns	ns	ns	-0.156*	-0.168*	-0.275**	-0.175*	-0.235**	ns	-0.290**	-0.228**
USMLE Step 1: 1st Attempt Pass	ns	ns	ns	ns	-0.270**	-0.303**	ns	ns	ns	ns	-0.256*
COMLEX-USA Level 2: 1st Attempt Pass	ns	ns	ns	ns	-0.228**	ns	ns	ns	ns	-0.154*	ns
USMLE Step 2: First Attempt Pass	-0.185^*^	-0.191^*^	ns	ns	ns	ns	ns	ns	-0.459^**^	ns	-0.203^*^
COMAT Failure: OB/GYN	ns	0.160^*^	ns	ns	0.168^*^	0.275^**^	0.235^**^	0.294^**^	0.194^**^	0.209^**^	0.224^**^
COMAT Failure: IM	ns	ns	ns	ns	0.159^*^	0.240^**^	0.215^**^	0.215^**^	0.190^**^	0.208^**^	0.184^*^
COMAT Failure: PSY	ns	ns	ns	ns	ns	ns	0.151^*^	ns	ns	ns	ns
COMAT Failure: SUR	ns	0.151^*^	ns	ns	ns	ns	ns	ns	0.152^*^	ns	ns
COMAT Failure: FM	ns	ns	ns	0.243^**^	0.313^**^	ns	ns	ns	ns	ns	0.226^**^

While pre-matriculation GPA, SGPA, and MCAT scores were weak predictors of board preparation course participation, they did not show significant predictive power for first-time pass rates of COMLEX-USA level 1. However, higher SGPA was weakly correlated with first-time COMLEX-USA level 2 pass rates. Performance in specific courses during the pre-clinical years, particularly in cardiology, neuroanatomy, and gastrointestinal courses, was highly predictive of first-time failure in COMLEX level 1 and level 2 exams. The strongest correlation with board preparation course participation was seen in endocrine/reproductive and gastrointestinal courses. Based on the findings, the Director of Academic Support at ACOM recommended targeted interventions, such as the introduction of small-group study sessions, mandatory academic support for students in the lowest quartile of specific courses, and education on the impact of sleep hygiene on academic performance. These interventions will be provided for the specifically identified courses, with the aim to improve student outcomes and reduce the likelihood of COMLEX-USA failures. These data provide a roadmap for ACOM to design proactive interventions to improve student success, particularly focusing on early identification of at-risk students and offering timely academic support.

## Discussion

The retrospective analysis of the ACOM’s Class of 2024 presents valuable insights into the predictive factors that influence first-time failures in the COMLEX-USA exams and participation in structured board preparation programs. These findings offer several key discussion points that contribute to the broader discourse on academic success predictors within osteopathic medical education.

The analysis found weak correlations between pre-matriculation metrics - such as GPA, SGPA, and MCAT scores - and the likelihood of first-time failure on COMLEX-USA level 1 and level 2 exams. Specifically, higher SGPA showed a weak but statistically significant correlation with first-time COMLEX level 2 success, while lower MCAT and SGPA scores were linked to a higher likelihood of needing board preparation. These results align with existing literature suggesting that, while pre-matriculation data can offer some insights into academic potential, it is not a definitive predictor of success in high-stakes medical licensing exams [5]. This suggests that, while admissions metrics remain important for selecting candidates likely to succeed in medical school, their predictive power diminishes when forecasting success in later stages, such as board examinations [5].

In contrast, post-admission performance, particularly in key pre-clinical courses such as cardiology, neuroanatomy, and gastrointestinal studies, showed much stronger correlations with negative outcomes. Students in the lowest quartile in these courses were significantly more likely to experience first-time failures on COMLEX-USA level 1 and level 2. This finding underscores the importance of continuous academic performance throughout the pre-clinical years as a more accurate predictor of future board exam success [6]. It also suggests that early identification of struggling students, especially those performing in the lowest quartile, could provide opportunities for timely intervention and support [7,8].

Based on the results, ACOM has implemented targeted interventions, including mandatory board preparation programs for students at risk of failing COMLEX exams and peer-led study groups focused on high-risk courses. The introduction of peer-led study groups supports findings in educational literature that peer learning and active group engagement can improve comprehension and retention, particularly in complex subjects [9]. Targeted interventions, including mandatory board preparation programs, were implemented to address identified academic challenges. The holistic approach also included sleep hygiene education to address the role of non-academic factors in academic performance.

The findings from ACOM’s retrospective analysis could serve as a model for other osteopathic medical schools facing similar challenges with first-time COMLEX-USA pass rates. While pre-matriculation metrics offer limited predictive power for exam success, continuous assessment and support during the pre-clinical years provide actionable data that institutions can use to intervene effectively. This shift from focusing on entry-level metrics to ongoing performance monitoring could significantly improve student outcomes and residency match rates across osteopathic medical schools.

While the study provides significant insights, it is limited by its single-institution scope, which may not be generalizable across other medical schools with different curricula or student demographics. Additionally, the study focuses primarily on academic predictors, and future research could explore non-academic factors - such as socioeconomic status, mental health, and extracurricular involvement - that may also impact student success. Longitudinal studies following students from matriculation through residency could further elucidate how these various factors interplay over the course of a medical career.

## Conclusions

In conclusion, the findings of this retrospective analysis of the ACOM's Class of 2024 provide significant insights into the predictors of student success on the COMLEX-USA level 1 and level 2 exams. While pre-matriculation data such as GPA, SGPA, and MCAT scores demonstrated weak correlations with first-time exam failure, participation in structured board preparation programs and post-admission performance, particularly in specific courses, emerged as more critical. Notably, students performing in the lowest quartile in courses such as cardiology, neuroanatomy, and gastrointestinal studies were found to be at a higher risk of failing the COMLEX-USA exams. This highlights the need for targeted academic interventions for at-risk students.

In response to these findings, ACOM has taken proactive steps to mitigate potential academic failures by enhancing academic support systems. Educating students about the correlation between course performance and board exam success, implementing peer-led study groups, and requiring individualized academic support for those in the fourth quartile are among the key strategies adopted. Additionally, the importance of sleep hygiene and study skills has been emphasized as part of a holistic approach to student success. These interventions, grounded in the data, aim to improve student outcomes and reduce the likelihood of first-time failures on the COMLEX-USA, thus enhancing students' competitiveness in the residency matching process. Future assessments will determine the efficacy of these interventions, ensuring that they are continually optimized to meet the evolving needs of ACOM students. This study is ongoing and will continue to analyze data relative to student performance over time.
